# Supplementary material for: Cyberbullying Perpetration and Socio-Behavioral Correlates in Italian and Spanish Preadolescents: A Cross-National Study and Serial Mediation Analysis
Source: Int J Environ Res Public Health. 2025 Mar 7;22(3):389. doi: 10.3390/ijerph22030389 (PMC11941868; doi:10.3390/ijerph22030389)
Supplement: Supplementary file 1 [file ijerph-22-00389-s001.zip › ijerph-3474788-supplementary.pdf]

International Journal of
Environmental Research
and Public Health

MDPI

SUPPLEMENTARY MATERIALS

Table S1. The English version of the BSMAS. Here are six statements to consider. For each, answer: (1) very rarely, (2) rarely, (3) sometimes, (4) often, or (5) very often.

1. You spend a lot of time thinking about social media or planning how to use it.	1	2	3	4	5
2. You feel an urge to use social media more and more.	1	2	3	4	5
3. You use social media in order to forget about personal problems.	1	2	3	4	5
4. You have tried to cut down on the use of social media without success.	1	2	3	4	5
5. You become restless or troubled if you are prohibited from using social media.	1	2	3	4	5
6. You use social media so much that it has had a negative impact on your job/studies.	1	2	3	4	5

Table S2. The English version of the MDS. The following items correspond to the various mechanisms of moral disengagement. Moral justification: 1, 9, 17, 25. Euphemistic language: 2, 10, 18, 26. Advantageous comparison: 3, 11, 19, 27. Displacement of responsibility: 5, 13, 21, 29. Diffusion of responsibility: 4, 12, 20, 28. Distorting consequences: 6, 14, 22, 30. Attribution of blame: 8, 16, 24, 32. Dehumanization: 7, 15 ,23, 31. Respondents indicate their agreement with each statement using a 5-point Likert scale, where: 1 = Strongly Disagree; 2 = Disagree; 3 = Undecided; 4 = Agree; 5 = Strongly Agree.

1. It is alright to fight to protect your friends.	1	2	3	4	5
2. Slapping and shoving someone is just a way of joking.	1	2	3	4	5
3. Damaging some property is no big deal when you consider that others are beating people up.	1	2	3	4	5
4. A kid in a gang should not be blamed for the trouble the gang causes.	1	2	3	4	5
5. If kids are living under bad conditions they cannot be blamed for behaving aggressively.	1	2	3	4	5
6. It is okay to tell small lies because they don't really do any harm.	1	2	3	4	5
7. Some people deserve to be treated like animals.	1	2	3	4	5
8. If kids fight and misbehave in school it is their teacher's fault.	1	2	3	4	5
9. It is alright to beat someone who bad mouths your family.	1	2	3	4	5
10. To hit obnoxious classmates is just giving them "a lesson."	1	2	3	4	5
11. Stealing some money is not too serious compared to those who steal a lot of money.	1	2	3	4	5
12. A kid who only suggests breaking rules should not be blamed if other kids go ahead and do it.	1	2	3	4	5
13. If kids are not disciplined they should not be blamed for misbehaving.	1	2	3	4	5
14. Children do not mind being teased because it shows interest in them.	1	2	3	4	5

15. It is okay to treat badly somebody who behaved like a "worm."	1	2	3	4	5
16. If people are careless where they leave their things it is their own fault if they get stolen.	1	2	3	4	5
17. It is alright to fight when your group's honor is threatened.	1	2	3	4	5
18. Taking someone's bicycle without their permission is just "borrowing it".	1	2	3	4	5
19. It is okay to insult a classmate because beating him/her is worse.	1	2	3	4	5
20. If a group decides together to do something harmful it is unfair to blame any kid in the group for it.	1	2	3	4	5
21. Kids cannot be blamed for using bad words when all their friends do it.	1	2	3	4	5
22. Teasing someone does not really hurt them.	1	2	3	4	5
23. Someone who is obnoxious does not deserve to be treated like a human being.	1	2	3	4	5
24. Kids who get mistreated usually do things that deserve it.	1	2	3	4	5
25. It is alright to lie to keep your friends out of trouble.	1	2	3	4	5
26. It is not a bad thing to "get high" once in a while.	1	2	3	4	5
27. Compared to the illegal things people do, taking some things from a store without paying for them is not very serious.	1	2	3	4	5
28. It is unfair to blame a child who had only a small part in the harm caused by a group.	1	2	3	4	5
29. Kids cannot be blamed for misbehaving if their friends pressured them to do it.	1	2	3	4	5
30. Insults among children do not hurt anyone.	1	2	3	4	5
31. Some people have to be treated roughly because they lack feelings that can be hurt.	1	2	3	4	5
32. Children are not at fault for misbehaving if their parents force them too much.	1	2	3	4	5

Table S3. The English version of the ECIP-Q, a 22-item self-report instrument designed to assess cyberbullying behaviors among adolescents. It evaluates two dimensions: cybervictimization and cyberaggression, each comprising 11 items. Respondents indicate the frequency of specific behaviors over the past two months using a 5-point Likert scale: 0 = Never; 1 = Once or twice; 2 = Once a month; 3 = Once a week; 4 = More than once a week.

1. Someone said nasty things to me or called me names using texts or online messages.	0	1	2	3	4
2. Someone said nasty things about me to others either online or through text messages.	0	1	2	3	4
3. Someone threatened me through texts or online messages.	0	1	2	3	4
4. Someone hacked into my account and stole personal information (e.g., through email or social networking accounts).	0	1	2	3	4
5. Someone hacked into my account and pretended to be me (e.g., through instant messaging or social networking accounts).	0	1	2	3	4
6. Someone created a fake account, pretending to be me (e.g., on Facebook or MSN).	0	1	2	3	4

7. Someone posted personal information about me online.	0	1	2	3	4
8. Someone posted embarrassing videos or pictures of me online.	0	1	2	3	4
9. Someone altered pictures or videos of me that I had posted online.	0	1	2	3	4
10. I was excluded or ignored by others in a social networking site or internet chat room.	0	1	2	3	4
11. Someone spread rumors about me on the internet.	0	1	2	3	4
12. I said nasty things to someone or called them names using texts or online messages.	0	1	2	3	4
13. I said nasty things about someone to other people either online or through text messages.	0	1	2	3	4
14. I threatened someone through texts or online messages.	0	1	2	3	4
15. I hacked into someone's account and stole personal information (e.g., through email or social networking accounts).	0	1	2	3	4
16. I hacked into someone's account and pretended to be them (e.g., through instant messaging or social networking accounts).	0	1	2	3	4
17. I created a fake account, pretending to be someone else (e.g., on Facebook or MSN).	0	1	2	3	4
18. I posted personal information about someone online.	0	1	2	3	4
19. I posted embarrassing videos or pictures of someone online.	0	1	2	3	4
20. I altered pictures or videos of another person that had been posted online.	0	1	2	3	4
21. I excluded or ignored someone in a social networking site or internet chat room.	0	1	2	3	4
22. I spread rumors about someone on the internet.	0	1	2	3	4
